# Prevalence of Extended-Spectrum Beta-Lactamase (ESBL)–Producing *Escherichia coli* in Humans, Food, and Environment in Kathmandu, Nepal: Findings From ESBL *E. coli* Tricycle Project

**DOI:** 10.1155/2024/1094816

**Published:** 2024-10-16

**Authors:** Jyoti Acharya, Runa Jha, Tulsi Ram Gompo, Sharmila Chapagain, Lilee Shrestha, Nisha Rijal, Anjana Shrestha, Pragya Koirala, Suraj Subedi, Binita Tamang, Hari Prasad Kattel, Bishal Khaniya, Basudha Shrestha, Aruna Karki, Ram Prasad Adhikari, Sanita Kayastha, Prasil Pradhan, Sarada Duwal Shrestha, Bijendra Raj Raghubanshi, Heera Tuladhar, Palpasa Kansakar, Saugat Shrestha, Priyanka Shrestha, Binay Shrestha, Ricardo J. Soares Magalhaes, Manish Kakkar, Arunkumar Govindakarnavar, Allison Gocotano, Reuben Samuel

**Affiliations:** ^1^National Public Health Laboratory, Tripura Marg, Kathmandu, Nepal; ^2^Central Veterinary Laboratory, Tripureshwor, Kathmandu, Nepal; ^3^Tribhuvan University Teaching Hospital, Maharajgunj, Kathmandu, Nepal; ^4^Kathmandu Model Hospital, Red Cross Marg, Kathmandu, Nepal; ^5^Nepal Medical College Pvt. Ltd., Jorpati, Kathmandu, Nepal; ^6^Patan Hospital, Patan Academy of Health Sciences, Satdobato Road, Lalitpur, Nepal; ^7^KIST Medical College Hospital, KIST Hospital Road, Lalitpur, Nepal; ^8^World Health Organization Country Office for Nepal, Kathmandu, Nepal; ^9^Queensland Alliance for One Health Science, School of Veterinary Science, The University of Queensland, Gatton, Australia; ^10^Children's Health and Environment Program, Children's Health Research Centre, The University of Queensland, Gatton, Australia; ^11^World Health Organization Southeast Asia Regional Office, New Delhi, India

## Abstract

The need to address antimicrobial resistance (AMR) through a One Health (OH) approach is now well recognized. There is, however, limited guidance on how AMR surveillance should be implemented across sectors to generate meaningful AMR and AMU data for decision-making. Using a sympatric approach to cross-sector sample collection, Nepal adopted the WHO extended-spectrum beta-lactamase (ESBL)–producing *Escherichia coli* (*E. coli*) Tricycle Project as a step toward OH surveillance for assessing the prevalence of ESBL-producing *E. coli* across human, veterinary, and environment sectors. This involved a three-stage approach: identification of human hotspots (Stage 1) and sample collection sites for poultry (Stage 2) and wastewater (Stage 3). A total of 53 blood cultures from patients with bloodstream infections (BSIs), 100 stool samples from healthy pregnant women, 220 poultry ceca from slaughterhouses and live markets, and 48 wastewater samples were processed for bacterial culture and analyzed for the presence of ESBL-producing *E. coli*. The prevalence of ESBL-producing *E. coli* among isolated *E. coli* was the highest in wastewater samples (91%) followed by human BSIs (49%), poultry (38.6%), and fecal carriage isolates from healthy pregnant females (15%). A statistically significant association was seen in the prevalence of multidrug resistance among ESBL producers (52%) and nonproducers (26%). ESBL-producing *E. coli* was detected in all wastewater samples tested except for the upstream river. The findings of the study showed a high prevalence of ESBL-producing *E. coli* in samples from all three sectors and provided baseline data based upon which strategies for the safe disposal of communal and hospital waste, integrated AMR surveillance, and control strategies could be planned and implemented.

## 1. Introduction

Antimicrobial resistance (AMR) remains a global challenge and imparts a significant health burden despite the increased focus and attention by the international community [1]. AMR reduces the effectiveness of the antimicrobial therapy and often increases the cost, incidence, and severity of infection. A study in 2019 conducted in 2019 estimated 4.95 million (3.62–6.57) deaths associated with bacterial AMR, including 1.27 million (95% UI 0.911–1.71) deaths attributable to bacterial AMR. If no action is taken to effectively prevent the continued emergence of AMR, this number is predicted to reach 10 million deaths per year by 2050, with the majority of these occurring in low- and middle-income countries. The AMR disease burden will be associated with an estimated economic cost of up to 100 trillion USD [2].

AMR is responsible for significant human and animal health impacts, and a One Health (OH) approach has been promoted to address the cross-system challenges of control and prevention. The lack of stewardship in antimicrobial use in the medical, veterinary, and agricultural sectors has been linked to the emergence of resistant pathogens [3]. Likewise, antibiotic residues, antibiotic-resistant bacteria (ARB), and antibiotic-resistant genes (ARGs) have also been recognized as emerging environmental pollutants that can spread rapidly across the globe [4].

Despite the growing worldwide attention given to AMR, there are substantial limitations to understanding the burden, distribution, and determinants of AMR at the population level. To understand and be prepared for the emerging AMR threats, it is necessary to monitor the rise and spread of resistant strains and lineages across all sectors. Among drug-resistant bacterial pathogens, extended-spectrum beta-lactamase (ESBL)–producing *E. coli* is a diverse and essentially ubiquitous microorganism that can readily cross barriers between humans, animals, and the environment and is considered a highly relevant global indicator of the magnitude of the AMR problem [5]. The global survey on ESBL-producing E. coli—“The Tricycle Protocol—a simplified, integrated trans-sectorial surveillance system for bacterial resistance to antibiotics on a global basis,” was developed by the World Health Organization (WHO) Advisory Group on Integrated Surveillance on Antimicrobial Resistance (AGISAR) and the Food Safety and Zoonoses Department of WHO. The proposed name “Tricycle,” after its three-wheeled namesake, is to demonstrate that it will simultaneously address three aspects of bacterial resistance (human health, food chain, and the environment) in a simple and feasible manner designed to provide robust, comparable, and valid statistical outcomes. It is hypothesized that if the spread and outbreak of ESBL-producing *E coli* can be controlled, then the global burden of AMR would be reduced [6].

A growing body of literature highlights the widespread distribution of resistant superbugs in Nepal [7]. Studies conducted in Kathmandu, Nepal, show a prevalence of 25%–33% ESBL-producing *E.coli* from clinical specimens including blood, 14.8% in poultry, and 16%–33.3% in water samples [8–13]. The need for an integrated AMR surveillance with an OH approach to demonstrate the connection between humans, the food chain, and the environment from an epidemiological perspective is hence well recognized. Nepal has also developed a National Action Plan to contain AMR, which emphasizes on the OH approach to monitor AMR trends [14]. In this regard, an attempt for AMR surveillance with the OH approach in the country was piloted through ESBL *E. coli* Tricycle Project with support from WHO, in 2021–2022. Nepal successfully completed the Tricycle Protocol involving human, poultry, and environment samples. In this paper, we describe the implementation and findings from the ESBL *E. coli* Tricycle Protocol in Nepal.

## 2. Methods

### 2.1. Study Design

The survey was implemented in Kathmandu, the capital city of Nepal, from November 2021 to December 2022, and the survey design was as per the WHO Global Tricycle Surveillance Protocol [6]. The city covers an area of 50.67 sq km and is inhabited by approximately 1,472,000 people in 2021 (https://worldpopulationreview.com/world-cities/kathmandu-population).

The sample collection and laboratory examination methods followed the standard Tricycle Protocol guidelines [6]. This involved sample collection from humans, poultry, and wastewater during the study period. An extension to the standard Tricycle Protocol was implemented to maximize the likelihood of detecting ESBL-producing *E. coli* across sectors and allow for the robust measurement of correlations in rates of ESBL-producing *E. coli* recovery across human, animal, and environmental sectors. In this context, site selection (across the three sectors) was based on a sequential, geospatial risk-based three-stage approach. In the first stage, past human ESBL-producing *E.coli* bloodstream notifications (up to 3 years of household geolocated data) in the main hospitals of Kathmandu were retrieved and mapped across the landscape of Kathmandu. Identification of historical clusters of ESBL-producing *E.coli* from bloodstream infections (BSIs) in humans was performed using Moran's I. In the second stage, poultry markets were identified within the historical clusters of human ESBL-producing *E.coli* clusters, in consultation with experts from the Ministry of Agriculture and Livestock Division (MoALD). A random sample of poultry markets within the historical human ESBL-producing *E. coli* clusters was selected for poultry sampling. In the third stage, wastewater sites were selected within the historical human ESBL-producing *E.coli* reporting hospitals, within river catchment sites with the clusters, and within poultry markets selected in Stage 2 of site selection.

#### 2.1.1. Human Sampling Protocol

A total of five hospitals, Tribhuvan University Teaching Hospital, Patan Hospital, KIST Hospital, Kathmandu Model Hospital, and Nepal Medical College Teaching Hospital, were included for human sample collection—for both cases and controls. All of these were tertiary care hospitals and were selected based on their data of patients with BSIs and performing > 5000 blood cultures in the previous year.

Sampling: Human samples consisted of case and control samples—blood samples for culture/susceptibility (referred to as case) from patients with BSI and stool samples (referred to as control) from healthy pregnant females in the third trimester of pregnancy visiting the same hospital for antenatal care (ANC). *E. coli* is the most frequent Enterobacteriaceae pathogen in the blood culture. Healthy pregnant women attending the hospital for routine antenatal checkups were included as control as this group represents healthy carriers who are less likely to have undergone antimicrobial treatment. As blood samples were collected from suspected bacteremia cases during routine clinical care, enrollment was not necessary. However, for control, the enrollment of participants was voluntary and with informed consent. Patients (both case and control) not residing in the Kathmandu Valley during the study period were excluded. No effort was made to actively collect samples for blood cultures (no purposive sampling).

#### 2.1.2. Poultry Sampling Protocol

Samples from poultry slaughterhouses and live bird markets were collected from Kathmandu and Lalitpur districts. Five different poultry live markets and slaughter areas were selected based on their spatial distributions within proximity to the human sampling sites. The broiler birds were chosen as representative of animal production because they could be a potential source of ESBL-producing *E. coli* in the food chain. The live broilers of marketable age were slaughtered, and their ceca were collected from the five selected market areas. Collection was done at a 1-month interval from the five markets in 11 events to minimize the sampling bias. Samples could not be collected in the month of February. Samples from one bird per vendor per stand at one point of time were collected to minimize the likelihood of the birds coming from the same farm or source.

Sampling: The intact ceca were obtained by clipping at the ileum and cecum junction and at the cecum and colon junction, and the entire cecum contents were placed in a sterile container. The cecum samples collected from freshly slaughtered broilers were deposited into a cooler and transported to the Central Veterinary Laboratory, Kathmandu, within 24 h for laboratory investigations.

#### 2.1.3. Environmental Sampling Protocol

For environment sampling, four sample types, namely, upstream, downstream, communal, and hospital effluent wastewater samples, were selected. Sites for environment sampling were selected based on the proximity to human sampling locations, effluent discharging from hospitals, and adjoining areas near poultry markets. For this, surface water samples were collected from six sites: one upstream river, one downstream river, three communal rivers that crossed through the sites for human and animal sampling, and hospital effluent samples from one of the hospitals included in the study. A total of eight wastewater samples were collected from each of these six sites at different time points during the study period. Surface water upstream of the city is representative of precity impacts and other upstream activities in the catchment area. It serves as a background sample to be able to detect the influence of the city population. Surface water downstream of the city is considered representative of city impacts, including human fecal waste discharges and other contributions from the city and its population. Communal rivers (collecting sewers) represented human discharges and as these also crossed poultry markets (or slaughterhouses if wet markets were not available) represented animal fecal material discharges. Hospital effluent from a major hospital represented microbiological contamination from the hospital population.

**Figure 1:**
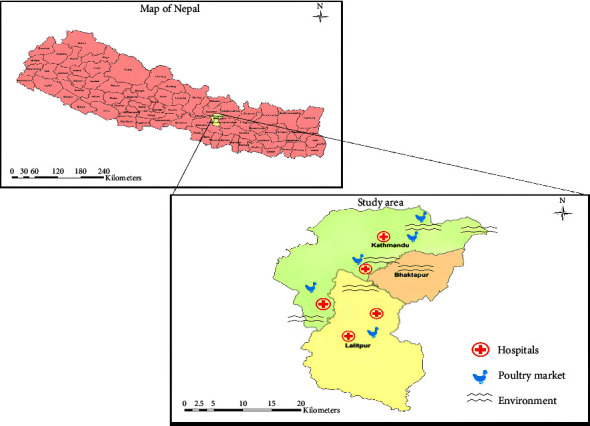
Map of Kathmandu showing the hospitals, poultry markets, and river sites for sample collection.

Sampling: Water samples were collected with a flow proportional sampling device. The sampling apparatus was rinsed a few times with the water sample. Three crude point samples, with at least a 5-min interval between each sampling, were collected. Samples were taken from beneath the surface (at 20–30 cm depth) and transferred with the funnel to the sample bottles. During transport to the lab, samples were stored at 2°C–8°C (in a cooling box) and processed within 24 h.  Figure 1 shows the sample collection areas for all three sectors.

A questionnaire tool was used to obtain epidemiological data from respondents in the human and animal sectors, and the characteristics of environmental samples were collected using a sample collection form.

### 2.2. Laboratory Isolation and Identification of *E. coli*

#### 2.2.1. Human Health

A total of 31,350 blood cultures were performed during the study period in the five hospitals according to the procedures established in each hospital (automated [BACTEC/Biomatrix] or conventional blood culture methods). Suspected blood culture-positive broths were subcultured on blood agar (MAST, Lot 500233, United Kingdom) and MacConkey (MAC) agar (MAST, Lot 500242, United Kingdom) plates, and lactose-fermenting colonies from positive blood cultures were selected and subcultured on MAC supplemented with cefotaxime 4 *µ*g/mL (MAC + CTX) (Sigma-Aldrich, Lot 077144773V Darmstadt, Germany), and the identification of *E. coli* was carried out using standard biochemical tests [15].

For control, 100 stool samples (20 from each hospital) were collected by trained professionals and transported in Cary Blair media to the laboratory for culture and identification. The samples were also plated on MAC supplemented with CTX 4 *µ*g/mL, and the identification of *E. coli* was carried out using standard biochemical tests.

Confirmed isolates (both case and control) were sent to the National Public Health Laboratory where reconfirmation of isolates was done by VITEK-MS, a matrix-assisted laser desorption ionization time-of-flight (MALDI-TOF)–based machine, before preservation at −75°C.

#### 2.2.2. Poultry

The cecum samples collected from the market-age broilers from five different markets were processed for bacterial culture at the Central Veterinary Laboratory. Around 5 g of cecum contents were taken out aseptically and added to 50 mL sterile buffered peptone water (BPW), incubated overnight, and then subcultured simultaneously onto MAC and MAC + CTX (MAC agar with CTX 4 *μ*g/mL). *E. coli* colonies were identified and confirmed by standard biochemical tests and MALDI-TOF. The *E. coli* isolates were screened for the ESBL production by the combination disk method (CDT).

#### 2.2.3. Environment

Forty-eight wastewater samples were collected from six sites during the study period, which covered all four seasons of Nepal. One hundred milliliters of wastewater collected from each site every month was transported to the National Public Health Laboratory within 24 h of collection for *E. coli* isolation, identification, enumeration, and test for ESBL production. Samples were processed following the direct one-step spread plate from serial dilutions of the sample (at different 10-fold dilutions) and membrane filtration method (undiluted and at different dilutions). Each dilution was plated on both tryptone bile X-glucuronide (TBX) agar (Oxoid, CM0945, Hampshire, England) and TBX bile X-glucuronide agar with 4 *µ*g/mL CTX (TBX agar + CTX). Presumptive *E. coli* colonies were counted and purified, and identification was confirmed by the standard biochemical identification tests and/or MALDI-TOF.

### 2.3. Antibiotic Susceptibility Testing

Antibiotic susceptibility testing of *E. coli* isolates from cases was done by the modified Kirby–Bauer disk diffusion method against ampicillin (10 *μ*g), ciprofloxacin (5*μ*), amikacin (30 *μ*g), gentamicin (10 *μ*g), ceftriaxone (CRO: 30 *μ*g), trimethoprim–sulfamethoxazole (1.25/23.75 *μ*g), amoxicillin–clavulanate (20/10 *μ*g), cefepime (30 *μ*g), and imipenem (10 *μ*g). The results were interpreted as per Clinical and Laboratory Standards Institute (CLSI), Performance Standards for Antimicrobial Susceptibility Testing, M 100-Ed32 [16].

### 2.4. Screening and Confirmation for ESBL Production Among *E. coli* Isolates

The initial screening test for the ESBL production was performed by using one of the three third-generation cephalosporin antibiotics: CRO 30 *μ*g, ceftazidime (CAZ: 30 *μ*g), or CTX 30 *μ*g disks. If the zone of inhibition (ZOI) was ≤25 mm for CRO, ≤22 mm for CAZ, and/or ≤ 27 mm for CTX, the isolate was considered a potential ESBL producer as recommended by CLSI (8). Isolates that were suspected as ESBL producers by the screening test were further confirmed by the CDT. For CDT, CAZ (30 *μ*g) alone and in combination with clavulanic acid (CAZ clavulanic acid, 30/10 *μ*g) disks were used. Isolates that showed an increase in the ZOI by ≥ 5 mm in the combination disks in comparison to that of either of the third-generation cephalosporin disks alone was considered an ESBL producer [16]. ATCC® *E. coli* 25922 was used as a negative control for ESBL production by disk diffusion and *Klebsiella pneumoniae* ATCC® 700603 as a positive control. All ESBL-producing *E. coli* isolates were stored in trypticase soy broth (TSB) with 20% glycerol at −80°C for further investigations.  Table 1 shows the characteristics of samples and ESBL-producing *E. coli* across all three sectors.

### 2.5. Data Analysis

Data analysis was performed using an Excel analysis tool pack, and statistical correlation was performed with the chi-square test where appropriate. A value of *p* < 0.05 was considered a cutoff for statistical significance.

### 2.6. Ethical Clearance

Ethical approval for this study was obtained from the Nepal Health Research Council, Government of Nepal (NHRC Registration No. 845-2019)

## 3. Results

The characteristics of pregnant women, patients with BSI, poultry from sellers/slaughterhouses, and environmental samples are presented in Table 2. The demographics of pregnant women showed that all control samples were from patients aged between 20–40 years and it was the first pregnancy for two-thirds of them. The patients with BSI were mostly from 19 to 60 years age group followed by the elderly (> 60 years).

A total of 57 vendors/slaughterhouses from the five poultry markets participated in the study to provide cecum samples. Most of these vendors or slaughter area workers (60%) had completed secondary-level education, but only 21% were trained in poultry meat handling. The majority of them (> 62%) were in the business for less than 10 years. Most vendors (> 50%) practiced chilling after slaughter by freezing if needed for storage for more than 24 h.

Of the 48 wastewater samples from six environmental sites, half were from the communal rivers. Environment samples were collected throughout the year representing all seasons.

### 3.1. ESBL-Producing *E. coli* in Human Population

Of the total 31,350 blood cultures performed at the participating hospitals, 2335 (7.5%) showed bacterial growth. *E. coli* comprised only 8.4% (195/2335) of the total bacterial isolates. Of the total 195 blood cultures positive for *E. coli*, only 53 fulfilled the inclusion criteria for human cases. Of these 53 *E. coli* isolates, 26 (49%) were ESBL producers. *E. coli* was isolated from all stool cultures of pregnant women (control samples) (100%), of which only 15 (15%) were ESBL producers. There was a significant difference between ESBL positivity among the cases and controls (*p* value < 0.001).

Among BSI cases, ESBL-producing *E. coli* was isolated mostly from the 19–60 years age group (12 out of 26), while only two isolates were from the pediatric age group (< 14 years). All ESBL-producing *E. coli* from controls were from 21–40 years age group.

The hospitalwise distribution of *E. coli* and ESBL-producing *E. coli* from case and control population is shown in Table 3. The isolation of *E. coli* from BSI cases among hospitals in the study varied from 5% to 22%. A statistically significant difference (*p* value < 0.05) for *E. coli* isolation was observed among the hospitals, with the highest isolation rate from Hospital C (22%) and the least from Hospital B (5%). Hospitals A and D reported the highest number of ESBL-producing *E. coli* from BSIs, while none were reported from Hospital E. The highest number of ESBL-producing *E. coli* from human controls were reported from Hospitals A and C, and none from Hospital D.

**Figure 2:**
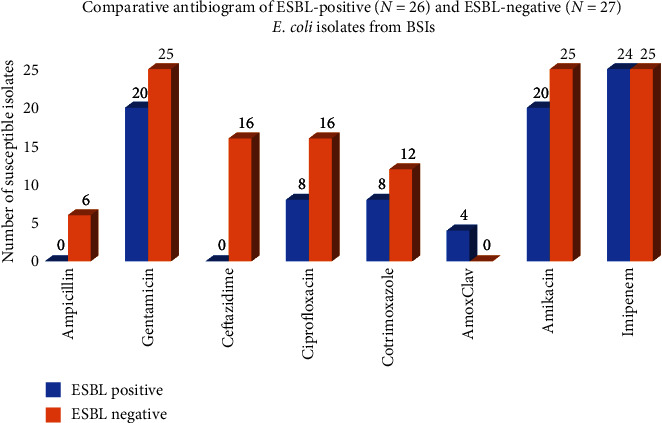
Comparative antibiogram of ESBL-producing and ESBL-nonproducing *E. coli* isolates from bloodstream infections. BSI, blood stream infection; ESBL, extended spectrum beta lactamase.

The antimicrobial susceptibility of ESBL-producing and ESBL-nonproducing *E. coli* isolates (from BSIs) is shown in Figure 2. ESBL-positive isolates from BSIs were susceptible to imipenem (24 out of 26), followed by amikacin and gentamicin (20 out of 26 each). More than half (52%) of the ESBL-positive isolates were multidrug resistant (MDR). ESBL-nonproducing *E. coli* from BSIs were susceptible to amikacin and imipenem (25 out of 27). Susceptibility among ESBL-nonproducing isolates to ciprofloxacin (16 out of 27) and cotrimoxazole (12 out of 27) was higher compared to ESBL-producing isolates (8 out of 26 for both ciprofloxacin and cotrimoxazole). Among ESBL-negative-*E. coli* isolates from BSIs, only 26% isolates were MDR. There was a significant association between the ESBL positivity and rate of MDR (*p* < 0.001).

### 3.2. ESBL-Producing *E. coli* From Poultry

A total of 220 cecum specimens were collected from 57 vendors of five live markets. The samples were collected in 11 events with 20 samples/event. The distribution of *E. coli* and ESBL-producing *E. coli* by market type and collection event is shown in Tables 4 and 5. *E. coli* was isolated from all 220 cecum samples tested, and about 39% of these were ESBL producers. The proportion of ESBL-producing *E. coli* ranged from 30% to 43% among the poultry sampling locations. It clearly varied between the sample collection events, ranging from 5% (II) to 100% (IX). Additionally, samples collected during the months of June and October had the highest positivity for ESBL-producing *E. coli* (70% and 75%, respectively), while the lowest during the months of March and April (5% and 15%, respectively).

Vendors/owners of slaughterhouses who received training on poultry slaughtering and meat handling had a comparatively low proportion of ESBL-producing *E. coli* (20%) in chickens compared to those who did not receive such trainings (44%). Similarly, vendors who had been in the business for a longer period (≥ 10 years) had a comparatively lower proportion of ESBL-producing *E. coli* (29%) in the chicken as compared to those with a shorter time in business (71%) although no significant (*p* > 0.05) association was noted for both scenarios.

Factors such as the source of water used (underground or tap) to clean the poultry, use of personal protective equipment while handling poultry, and method of chilling the poultry and cleaning the stall did not show any significant association with ESBL positivity (*p* > 0.05). However, a borderline statistically significant difference was noted between the sites of origin of poultry (i.e., supplied from outside (55%) versus within the Kathmandu Valley (45%) (*p* < 0.05)).

### 3.3. Characteristics of Samples and ESBL-Producing *E. coli* From Environment Sector

*E. coli* was present in 43 out of 48 wastewater samples tested (90%). The prevalence of ESBL producers among water samples cultured was 81% (39/48 samples) and 91% among total *E. coli* isolates (39/43 *E. coli* isolates). Five samples in which *E. coli* was not detected were from the upstream river (Mulkharka). The proportion of ESBL-producing *E. coli* was higher in the monsoon/autumn season (Aug–Oct) as compared to the spring and summer, and least in the winter (Nov–Jan). The seasonal and sitewise distribution of the average concentration of *E. coli* and ESBL-producing *E. coli* and the proportion of ESBL-producing *E. coli* from environmental samples are given in Table 6. All sites except the upstream river showed the presence of ESBL-producing *E. coli* with varying concentrations (1.8 × 10^5^–6 × 10^6^ CFU/100 mL) at different events of collection. Upstream wastewater samples showed the lowest concentration of *E. col*i, and a gradient increase was seen in the *E. coli* concentration in communal rivers through upstream to downstream.

Among the communal effluents, the highest concentration of *E. coli* was found in Sobha Bhagwati (7.1 × 10^6^), but the highest concentration of ESBL-producing *E. coli* was seen in Dhobikhola (9.5 × 10^5^). Surprisingly, the hospital effluent showed the lowest concentration of both *E. coli* and ESBL-producing *E. coli* (1.8 × 10^6^ and 1.8 × 10^5^, respectively). The ratio of ESBL-producing *E. coli* to total *E. coli* was found lowest in downstream river samples (Jal Binayak River).

## 4. Discussion

In this study, we analyzed the prevalence of ESBL-producing *E. coli,* which is considered an OH indicator organism for antibiotic resistance, considering the similar temporal scale and sympatric, risk-based geographical interfaces. To the best of our knowledge, this is the first integrated AMR surveillance study encompassing human health, animal health, and environmental sector in Nepal. *E coli* was seen in all human control and poultry cecum samples but only in 90% of environment water samples. In blood cultures, *E. coli* constituted only 8% of total culture positives. ESBL-producing *E. coli* were detected in samples collected from all three sectors. For human cases, *E. coli* isolates meeting the study criteria were only included and processed further for ESBL detection. Hence, data on ESBL-producing *E. coli* out of the total blood samples cultured were not available. Of the total samples cultured, ESBL-producing *E. coli* were isolated from 39% (85/220) of the poultry and 81% (39/48) of environmental samples, whereas the proportion of ESBL producers among *E. coli* isolates was 49% (26/53) in human cases, 15% (15/100) in human controls, 39% (85/220) in poultry, and 91% (39/43) in environmental samples.

*E. coli* is the most common gram-negative pathogen that causes BSI [17]. In our study, the prevalence of *E. coli* and ESBL-producing *E. coli* in BSIs was 8.3% and 49%, respectively. Although the total *E. coli* isolated from BSIs was higher, isolates from patients failing to comply with the inclusion criteria and without filled questionnaires were excluded. Our findings are in line with studies conducted in Nepal, which show a prevalence of 8.6% and 13% *E. coli* with 30% and 45.4% ESBL-producing *E. coli*, respectively [18, 19].

In our study, the prevalence of ESBL-producing *E. coli* in healthy pregnant women was 15%, which to our knowledge is a new finding for Nepal. Our results are similar to a study conducted in India [20], which showed 15% ESBL-producing *E. coli* isolates among pregnant females, but were lower than in other studies conducted in Tanzania [21] and Indonesia [22], which showed a higher prevalence of ESBL-producing *E. coli* (64% and 31%, respectively). A similar pilot study of ESBL-producing *E. coli* in Indonesia [23] also showed 40% prevalence. The variation with other studies could be due to the study population being taken from primary health centers rather than from tertiary care institutions. Other contributing factors may be the level of education, poor sanitation and hygiene in hospitals, food chains that allow for the transmission of resistant strains, and varying geographical locations.

The isolation of ESBL-producing *E. coli* from BSIs was higher from patients of 19–60 years age group followed by the elderly (> 60 years age group), which is in concordance with a pilot study from Indonesia [23]. Studies showed that BSI in extreme ages (either neonate or elderly) is common and fatal [24, 25]. In our study, pediatric infections were very low. This may be because none of the selected study sites was a pediatric hospital; therefore, the representation of pediatric samples may have been inadequate.

Regarding the antibiotic susceptibility of *E. coli* among BSI cases, ESBL-nonproducing isolates were more susceptible than ESBL producers to the tested antibiotics except for gentamicin and imipenem. This discrepancy may be due to the very low number of isolates from BSIs in this study (only 53). The ratio of MDR isolates among ESBL producers versus ESBL nonproducers was 2:1 in our study. Although high MDR rates among ESBL isolates have been reported in other studies from Gaza (92% in ESBL producers vs. 60.8% in non-ESBL producers), the difference is not striking unlike in the present study (*p* < 0.05) [26].

In the poultry samples, we found 100% (220/220) prevalence of *E. coli* in ceca of chicken, which is in accordance with similar studies done in Kathmandu and nearby areas [27, 28] but higher than that reported in Indonesia (78.8%) [23]. It is because *E. coli* are the commensal organisms in the poultry gut. On the other hand, the prevalence of ESBL-producing *E. coli* in our study was 39%, which was higher than those reported by Kharel et al. (14.81%) in a study done in Kirtipur poultry farms in Nepal, but almost half as reported by a pilot study in Indonesia (67%) [11, 23]. The higher proportion of ESBL-producing *E. coli* in food chains can be due to many factors, including the use of antibiotics in livestock and poultry and contamination of livestock production environments such as feed and/or water with ESBL-producing *E. coli* [29]. Although there was no significant difference in the proportion of ESBL-producing *E. coli* by sampling sites (30% to 43%), a variation was observed among sample collection events, ranging from 5% (in Event II) to 100% (in Event IX). It is interesting to note that the high percentage of ESBL-producing *E. coli* in the food chain does not always correlate with the frequency of ESBL-producing *E. coli* in BSIs. Similar observations were made in the Netherlands in some studies [30, 31]. However, exact associations can be underpinned by the help of further genomic analysis of the *E. coli* isolates.

The prevalence of *E. coli* and ESBL-producing *E. coli* among 48 environmental samples was 90% and 81%, respectively, in our study, which is higher than those conducted in other parts of Nepal—Dharan (44% *E. coli* and 43% ESBL-producing *E. coli*) [32] and Biratnagar (70% *E. coli* and 57% ESBL-producing *E. coli*) [33]. This difference may be because the latter studies were done in effluents and hospital waste only. A similar study from Bangladesh showed 100% *E. coli* and 50% ESBL producers [5], whereas a study from Pakistan revealed 65% *E. coli* and 71% ESBL producers in surface and wastewater samples [34]. We observed that water from the upstream river showed the presence of *E. coli*, though no ESBL producers. Communal wastewater samples had varying concentrations of both *E. coli* and ESBL-producing *E. coli* with a gradient increase in the *E coli* concentration from upstream to downstream samples. The ratio of concentration of ESBL-producing *E. coli* to total *E. coli* was found lowest in downstream river samples, which could be a result of the “dilution effect.” In the Kathmandu Valley, river water is also used for irrigation purposes, which raises concern on the transmission of drug-resistant microbes from the water into the vegetables and food crops, which could infect humans through the food chain [35]. Similarly, in hospital wastewater, the concentration of both *E. coli* and ESBL-producing *E. coli* was lower than that of communal wastewater, which may be because of the various kinds of detergents and disinfectants used in the hospitals.

The seasonal distribution showed a high proportion of ESBL-producing *E. coli* in the August to October season as compared to others. This result differs from other studies conducted in Germany [36], which stated a higher load of ESBL-producing *E. coli* in winter, in Indonesia [23], which stated that the rainy season had a higher load, and in China, which showed that ESBL-producing *E. coli* isolates were detected most frequently in samples collected in the spring. In Nepal, August to October is the season of major festivities, during which there is mild rainfall and animal slaughtering and disposing is the highest, which may be a reason behind the high concentration of *E. coli* in wastewater samples.

The results of the present study demonstrated that a geospatially guided risk-based surveillance design is a sensitive approach to estimating gradients of ESBL-producing *E. coli* across sectors and is a robust approach for the monitoring and evaluation of AMR-reducing interventions in the community. In Nepal, AMR surveillance and containment efforts have started to gain momentum as one of the public health priorities by the Ministry of Health and Population as well as other quadripartite ministries. The implementation of the national action plan on AMR includes strategies and policies to promote good husbandry practices and a coordinated OH surveillance on AMR and antimicrobial use and to raise awareness among producers and consumers on issues of AMR and on safe disposal of hospital and communal water. Moreover, the irrational use of antibiotics and the illegal importation of medicines need to be strictly controlled. There is a well-established laboratory network for the detection of AMR in the human and veterinary sectors, while the food and environment sectors are gradually building up their capacity for AMR surveillance. This study was a model for the AMR surveillance in the OH approach in Nepal and is expected to be conducted by expanding the coverage area gradually. The results from this study could be used as the baseline data for integrated action from all three sectors to address AMR. Although there is good interministerial coordination among sectors, the lack of a dedicated budget, inconsistent availability of resources (such as media and reagents), and shortage of well-trained and efficient workforce in these sectors are the challenges in its long-term implementation.

## 5. Conclusion

Our study showed a high prevalence of ESBL-producing *E. coli* in humans, poultry, and environment samples. The findings from this study calls for the urgent need for interventions in the OH approach to prevent the spread of antibiotic-resistant pathogens. The interventions in Nepal for controlling AMR have mainly focused on managing the use of antibiotics in humans and animals. The high prevalence of ESBL-producing *E. coli* from water samples suggests the need for interventions for improved wastewater management and sewerage treatment to prevent environmental contamination as a potential source for AMR and disease outbreaks. We also recommend educating the general population on reducing the practice of discharging untreated waste into water bodies, open defecation, and the risks of using such contaminated water for domestic purposes. Education should also focus on food handlers including poultry meat vendors on improving the working environment and hygienic practices, as well as periodic screening for colonization with ARB.

Further investigations are recommended to understand the genetic relatedness, sources, and transmission pathways of the ESBL-producing *E*. *coli* isolates. Additional genomic characterization of the ESBL-producing *E*. *coli* isolated from the three sectors would provide the distribution and diversity of elements encoding ESBL and other AMR determinants.

This study was conducted as a pilot project, and we recommend the expansion of similar studies in other geographical regions of Nepal to get a nationally representative picture of the ESBL-producing *E. coli* distribution. The Tricycle surveillance model can also be adopted as an ongoing surveillance; however, this needs consideration in simplifying the questionnaire so that it is filled properly.

## 6. Limitations

The present study has several limitations. First, in human cases, questionnaires were only collected from patients whose blood culture yielded ESBL-producing *E. coli*. This led to a situation where back tracing of patients and questionnaires could not be completed due to loss of contact. The protocol should emphasize on collecting information from all eligible BSI patients with *E. coli* regardless of ESBL positivity. The exclusion of isolates from patients who did not reside within the valley further reduced the number of samples. Second, the Tricycle Protocol requires testing three to five different colonies per sample (for human control, poultry, and environment), and despite these being the predominant isolates within a sample, they may not represent the total genetic diversity of ESBL-producing *E. coli*.

## Figures and Tables

**Table 1:** Outline of sample collection and processing of various samples for detection of ESBL-producing *E. coli* across all three sectors during the ESBL *E. coli* Tricycle Project in Nepal from November 2021 to December 2022.

Variable	Human sector		Animal sector	Environmental sector
	Control	Case		
Sample	Stool/rectal swab from healthy pregnant women	Blood culture from patients with suspected bloodstream infection	Poultry ceca	River/communal wastewaters and hospital effluent

Number of samples	100	53	220	48

Sampling site	Five hospitals from selected area	Five hospitals from selected areas	Five markets from selected areas	Six rivers/effluent sites from selected areas

Epi data	Yes	Yes	Yes	Yes

Laboratory	Sample processing: At selected hospital sites Reconfirmation: National Public Health Laboratory	Sample processing: At selected hospital sites Reconfirmation: National Public Health Laboratory	Sample processing: Central Veterinary Laboratory Reconfirmation: National Public Health Laboratory	Sample processing and reconfirmation: National Public Health Laboratory

Culture media used	MAC and MAC + CTX	MAC and MAC + CTX	Buffered peptone water, MAC and MAC + CTX	TBX and TBX + CTX agar

E. coli identification	Biochemical tests Reconfirmation: MALDI-TOF	Biochemical tests Reconfirmation: MALDI-TOF	Biochemical tests Reconfirmation: MALDI-TOF	Biochemical tests Reconfirmation: MALDI-TOF

Antibiotic susceptibility tests (ASTs)	NA	Modified Kirby–Bauer's disk diffusion	NA	NA

ESBL confirmation	Combination disk test (CDT)	Combination disk test (CDT)	Combination disk test (CDT)	Combination disk test (CDT)

Abbreviations: CTX, cefotaxime; ESBL, extended spectrum beta lactamase; MAC, MacConkey Agar; MALDI-TOF, Matrix-Assisted Laser Desorption Ionization Time of Flight; TBX, Tryptone Bile X-Glucuronide Agar.

**Table 2:** Sample characteristics for human, poultry, and environment sectors.

Variable	Number (*n*)	Percentage (%)
A. Human control: pregnant women (*N* = 100)		
Age in years		
21–30	43	43.0
31–40	57	57.0
Pregnancy		
Primigravida	66	66.0
Multigravida	34	34.0
B. Human cases of BSI (*N* = 53)		
Age in years		
< 5	5	9.4
5–18	4	7.5
19–60	24	45.2
> 60	20	37.7
Sex		
Male	18	33.96
Female	35	66.04
Comorbidities		
Yes	16	30.1
No	37	69.8
C. Animal sector: characteristics of vendors at the market or the slaughterhouse (*N* = 220)		
Vendors		
Male	182	82.7
Female	38	17.3
Education		
No schooling	19	8.6
Primary/secondary level	133	60.4
Higher secondary/university level	68	31
Years in business		
< 10 years	137	62.2
> 10 years	83	37.8
Training received for poultry processing		
Yes	46	21
No	174	79
Process of chilling after slaughter		
No chilling	40	18.2
Kept in freezer if > 24 h	114	51.8
Dry chilling (using cold water)	66	30
D. Environment		
Sample type		
River upstream	8	16.7
Communal (three different rivers)	24	50.0
Hospital effluent	8	16.7
River downstream	8	16.7
Season		
Winter (Nov–Jan)	14	29.2
Spring (Feb–Apr)	13	27.1
Summer/monsoon (May–July)	13	27.1
Monsoon/autumn (Aug–Oct)	8	16.7

Abbreviation: BSI, blood stream infection.

**Table 3:** Comparison of ESBL-producing *E. coli* from human cases and controls based on hospital.

Variable	Sites (hospitals)					
	A *N* (%)	B *N* (%)	C *N* (%)	D *N* (%)	E *N* (%)	Total
Case						
Total number of blood cultures	13,109	8,635	3,227	3,154	3,225	31,350
Blood culture positive, number (%)	746 (5.6%)	1,257 (14.5%)	64 (1.98%)	138 (4.3%)	130 (4.0%)	2,335 (7.4%)
Prevalence of *E. coli* among culture positives, number (%)	85/746 (11%)	57/1,257 (5%)	14/64 (22%)	27/138 (20%)	12/130 (9%)	195/2,335(8%)
*E. coli* included in the study out of total *E coli* isolates, number (%)	17/85 (20%)	6/57 (11%)	5/14 (35%)	16/27 (59%)	9/12 (75%)	53/195 (27%)
ESBL-producing *E. coli* out of total *E. coli* included in the study, number (%)	9/17 (53%)	4/6 (67%)	4/5 (80%)	9/16 (56%)	0	26/53 (49%)
Control						
Stool samples	20	20	20	20	20	100
*E. coli* isolated among total stool samples cultured, number (%)	20/20 (100%)	20/20 (100%)	20/20 (100%)	20/20 (100%)	20/20 (100%)	100/100 (100%)
ESBL-producing *E. coli* among total *E. coli* isolated, number (%)	5/20 (25%)	2/20 (10%)	5/20 (25%)	0	3/20 (15%)	15/100 (15%)

Abbreviation: ESBL, extended spectrum beta lactamase.

**Table 4:** Distribution of ESBL-producing *E. coli* by different sampling locations.

SN	Marketplace	Number of samples	*E. coli*, n (%)	ESBL-producing *E. coli* out of total sample and total *E.coli* isolates *n* (%)
1	A (Chabahil)	47	47 (100)	19 (40.4)
2	B (Chandragiri)	44	44 (100)	19 (43.2)
3	C (Jorpati)	41	41 (100)	19 (43.1)
4	D (Sobha Bhagwati)	36	36 (100)	13 (29.5)
5	E (Lagankhel)	52	52 (100)	15 (36.6)
	Total	220	220 (100%)	85 (38.6%)

Abbreviation: ESBL, extended spectrum beta lactamase.

**Table 5:** Monthly distribution of ESBL-producing *E. coli* in the poultry market and slaughterhouses (food chain).

Sampling event (month)	Number of samples tested	*E. coli*, *n* (%)	ESBL-producing *E. coli* out of total sample and total *E.coli* isolates *n* (%)
I (January)	20	20 (100)	6 (30)
II (March)	20	20 (100)	1 (5)
III (April)	20	20 (100)	3 (15)
IV (May)	20	20 (100)	8 (40)
V (June)	20	20 (100)	14 (70)
VI (July)	20	20 (100)	6 (30)
VII (August)	20	20 (100)	8 (40)
VII (September)	20	20 (100)	7 (35)
IX (October)	20	20 (100)	15 (75)
X (November)	20	20 (100)	8 (40)
XI (December)	20	20 (100)	9 (45)
Total	220	220 (100%)	85 (38.6%)

**Table 6:** The average *E. coli* concentration, ESBL-producing *E. coli* concentration, and percentage of ESBL-producing *E. coli* from environmental samples based on sampling sites and sampling events.

Source	Number of samples	Number positive for *E. coli*	Number of ESBL-producing *E.coli*	Average		
				*E. coli* concentration (CFU/100 mL) (A)	ESBL-producing *E. coli* concentration (CFU/100 mL) (B)	% ESBL-producing *E. coli* (B/A)
A (upstream: Mulkharka)	8	3	0	2.7 × 10^3^	0	0
B (communal: Manohara)	8	8	7	2.2 × 10^6^	2.1 × 10^5^	9.3
C (communal: Dhobikhola)	8	8	8	6.5 × 10^6^	9.5 × 10^5^	14.5
D (communal: Sobha Bhagwati)	8	8	8	7.1 × 10^6^	7.9 × 10^5^	11.1
E (downstream: Jal Binayak)	8	8	8	3.6 × 10^6^	3.1 × 10^5^	8.7
F (hospital effluent)	8	8	8	1.8 × 10^6^	1.8 × 10^5^	10.2
Event						
I: winter (Nov–Jan)	14	12	11	2.7 × 10^6^	1.4 × 10^5^	5.2
II: spring (Feb–April)	13	11	11	6.5 × 10^6^	7.3 × 10^5^	11.2
III: summer/monsoon **(**May–July)	13	12	11	3 × 10^6^	4.8 × 10^5^	15.7
IV: monsoon/autumn (Aug–Oct)	8	8	6	1.2 × 10^6^	2.3 × 10^5^	19.2

Abbreviation: ESBL, extended spectrum beta lactamase.

## Data Availability

The data that support the findings of this study are available from the corresponding author, Palpasa Kansakar, upon reasonable request.
